# IR Action Spectroscopy of Mass- and Mobility-Selected α‑Synuclein Segments: Structure Identification in Early-Stage Oligomers via the Amide A Region

**DOI:** 10.1021/acs.jpca.5c02819

**Published:** 2025-08-21

**Authors:** Agathe Depraz Depland, Olivier Verhoev, Steven Daly, Anouk M. Rijs

**Affiliations:** † Division of Bioanalytical Chemistry, Department of Chemistry and Pharmaceutical Sciences, Amsterdam Institute of Molecular and Life Sciences, 1190 Vrije Universiteit Amsterdam, De Boelelaan 1105, 1081 HV Amsterdam, The Netherlands; ‡ Centre for Analytical Sciences Amsterdam (CASA), Amsterdam, The Netherlands; § MS-Vision, Televisieweg 40, 1322 AM Almere, Netherlands

## Abstract

Parkinson’s disease (PD) is associated with protein misfolding and aggregation of α-Synuclein (α-Syn), a process central to its pathology. Understanding the early structural transitions of α-Syn is crucial, yet detailed insights into oligomeric intermediates remain elusive. Using the novel Photo-Synapt spectrometer, which integrates ion mobility mass spectrometry (IM-MS) with infrared multiple-photon dissociation (IRMPD) spectroscopy, we investigated the secondary structures of key α-Syn peptide segments, namely, WT-PD1 (NACore) and WT-PD2 (pre-NAC). This unique approach enabled conformationally selective analysis, allowing peptide monomers and oligomers to be separated based on both mass and mobility. Despite sequence similarities, these peptides exhibit distinct monomeric structures and aggregation behavior, leading to different oligomer assemblies with unique IR signatures. By analyzing the amide A region (3100–3500 cm^–1^), we provide structural insight into α-Syn oligomer structure, including the resolution of parallel versus antiparallel β-sheets and the identification of non-β motifs such as γ-turns. We further examined the G51D mutation from the pre-NAC region (G51D-PD2), which induces a more compact structure and stabilizes oligomers, leading to distinct IR spectral features. Combining IM-MS and IRMPD spectroscopy within a single instrument provides a novel structural readout for early-stage peptide aggregation. This approach highlights the high sensitivity of sequence-dependent structural transitions in α-Synuclein oligomers, providing insight into the mechanistic pathways of sequence-dependent folding relevant to disease.

## Graphical abstract

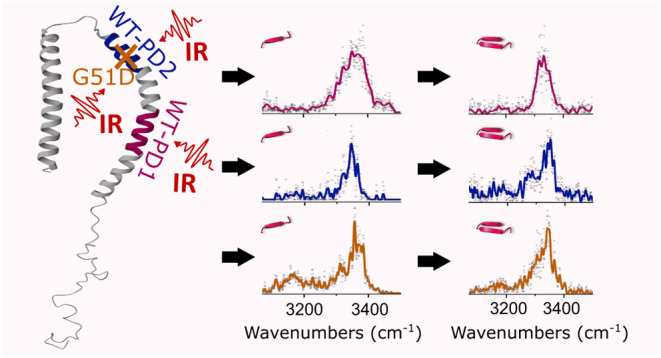

## Introduction

Parkinson’s disease (PD) is a progressive neurodegenerative disorder characterized by the formation of intracellular protein aggregates, known as Lewy bodies, which are associated with the loss of dopaminergic neurons.^1^ The primary component of these aggregates is α-Synuclein (α-Syn), a protein whose misfolding and aggregation are central to the pathology of the disease.^2–4^ Both early-stage, transient oligomers formed along the aggregation pathway as well as the resulting mature amyloid fibrils are implicated in toxicity; however, the early-stage oligomers are considered the more toxic species.^5–8^ Here, we focus on the oligomer formation of a key segment of α-Syn, known as the NACore (residues 68–78), which is the minimal entity that contains all of the features of aggregation and toxicity of the full-length protein.^9–12^ Variations and mutations in α-Syn, particularly in the pre-NAC region (residues 47–56),^11–16^ have been associated with PD. One example is the G51D mutation, where an apolar amino acid is replaced by an acidic amino acid. This mutation has been shown to slow down the aggregation of α-Syn compared to the wild type, while being associated as well to the early onset PD.^14,17^ The aggregation of α-Syn peptide segments involves the formation of a variety of oligomeric species with distinct conformations and secondary structures, which complicates structural characterization.

Ion mobility mass spectrometry (IM-MS) has emerged as a powerful tool for separating and identifying different conformers of peptides and proteins and of ions possessing a singular *m*/*z* value, such as [*n*M + *n*H]^*nz*+^ oligomers with *n* being the number of monomeric units and *z* being the charge.^18–27^ Although IM-MS provides valuable information about the overall shape and size of the aggregates, it does not directly resolve secondary structural information on peptides. Infrared (IR) spectroscopy provides this level of detail. Typically, the stretching mode of C=O groups (amide I) of the peptide backbone is used, which can be especially diagnostic for different types of secondary structures.^28–30^ Highly ordered structures, such as α-helices and β-sheets, yield major amide I bands at 1650–1660 cm^–1^ and 1610–1640 cm^–1^, respectively.^31–33^ However, conventional IR spectroscopy is limited when applied to complex samples, as it lacks isomer and/or oligomeric specificity. Hyphenation of infrared multiple-photon dissociation (IRMPD) spectroscopy with IM-MS allows for the isolation of individual oligomers, isomers, and/or conformers, for example, and offers enhanced specificity to probe secondary structural features.^31,34–39^ Although IR spectroscopy in the amide I region (C=O stretch from 1600 to 1700 cm^–1^) is widely used to probe β-sheets in peptide assemblies,^31,40^ distinguishing specific structural signatures can be challenging due to overlapping vibrational modes.^41–43^ While it enables the classification of parallel versus antiparallel β-sheets, resolving finer structural details within the sheets remains difficult.^40^

To complement this approach, we investigate the amide A region (3100 and 3500 cm^–1^) of the IR spectrum, where NH stretching vibrations may provide an additional insight into β-sheet structures and other secondary motifs.^40,44–46^ By coupling IRMPD spectroscopy with IM-MS, we use the strengths of both techniques, namely, IM-MS, for separating and identifying oligomers and IRMPD spectroscopy for detailed structural characterization. This integrated approach enables a better understanding of the secondary structures of transient early onset oligomers and their transitions into organized β-sheet structures. We measured the IR signatures of _68_GAVVTGVTAVA_78_ (WT-PD1 from the NAC-core), _47_GVVHGVATVA_56_ (WT-PD2 from the pre-NAC), and the PD2 mutant _47_GVVHDVATVA_56_ (G51D-PD2) oligomers; see Scheme 1A–C, respectively. Using a qualitative, comparative approach, this study explores the potential of the amide A region to provide more precise insights into β-sheet formation in the aggregation of peptides from α-Synuclein relevant to Parkinson’s disease.

**Scheme 1:**
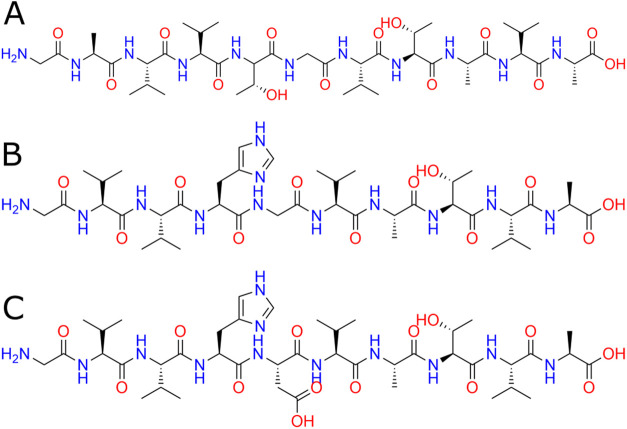
Chemical Structure of (A) Wild-Type _68_GAVVTGVTAVA_78_ (WT-PD1) Segment from the NAC Region, (B) Wild-Type _47_GVVHGVATVA_56_ (WT-PD2) Segment from the Pre-NAC Region, and (C) Mutant _47_GVVHDVATVA_56_ (G51D-PD2) Peptide Segment from the Pre-NAC Region of the α-Synuclein Protein

## Methods

### Sample Preparation

The NAC, the pre-NAC, and the mutated peptide segments from α-Synuclein, _68_GAVVTGVTAVA_78_ (WT-PD1), _47_GVVHGVATVA_56_ (WT-PD2), _47_GVVHDVATVA_56_ (G51D-PD2), and _47_GVVHGVTTVA_56_ (A53T-PD2), were purchased from Biomatik and used without further purification (>95%). After aliquoting with 1,1,1,3,3,3-hexafluoro-2-propanol (HFIP),^19,21^ the samples were left to dry overnight and then stored at −20 °C. Each peptide stock solution was prepared from the aliquots, using a peptide-specific protocol in order to initiate the aggregation. **WT-PD1 and WT-PD2 peptides**: a stock solution of 100 μM was prepared in a 10 mM ammonium acetate (AA) solution (Merck) at pH 7.4. **G51D-PD2:** a stock solution of 1 mM was prepared in a 10 mM AA solution with pH 7.4 for peptides.

Following dissolution of the samples (WT-PD1, WT-PD2, and G51D-PD2), the solution was shortly vortexed (about 5 s), subsequently sonicated for 20 min, and incubated at 37 °C. All peptide samples were further diluted to 20 μM for nano-ESI infusion in positive mode in the Photo-Synapt.^18,19^ Random time points were measured over the course of 24 h after sample preparation as oligomers are typically formed within the first hours after dissolution of the peptide.

### Ion Mobility Mass Spectrometry Conditions

The Photo-Synapt,^18^ a customized ion mobility mass spectrometer with mobility slicing capabilities and optical access, was operated in positive ion mode with mobility turned on. The voltage applied to the nanotip capillary varied between 0.5 and 1.4 kV to be set to the minimal possible voltage allowed while keeping a stable signal. This allowed us to preserve the structure of the fragile assemblies depending on the cut performed on the tip as well as on the ions present at each time. Mobility separation was performed at wave velocity 260 m s^–1^ and wave height 11.0 V on quadrupole-selected values of *Q* 944.5 *m*/*z* and *Q* 909.5 *m*/*z* for WT-PD1 and WT-PD2, respectively. Once the arrival time spectra (also referred to as mobility spectra or mobilograms) were measured, peaks were identified and sliced according to their arrival times. Each slice was subsequently trapped and irradiated with a tunable IR laser with irradiation times as presented in Table S1. The resulting fragment ions were detected using the mass analyzer. This procedure has been described in detail by Bakels et al.^18^

### IRMPD Spectroscopy Conditions

The tunable IR laser (FireFly IR, M-Squared) was set to scan from 2800 to 3500 cm^–1^, with a repetition rate of 150 kHz, a pulse width of 10 ns, and an output power of about 80 mW over the full IR range. The trapping conditions for each mobility peak, the irradiation times, and for each region of the IR spectrum are described in Table S1. The irradiation times for monomers are significantly longer than those for higher-order oligomers. For the latter, multiple-photon IR absorption results in the monomer release by dissociation of hydrogen bonds. For the monomer, however, b/y fragmentation occurs via the cleavage of the peptide bonds.

### Data Processing

MassLynx V4.1 (MassLynx software instrument control) was used to obtain the mass and mobility data, and Origin 2023 was used to analyze and plot the data. The ion mobility spectra presented for each peptide are averaged over all of the recorded mobility spectra from which mobility peaks were selected, sliced, and trapped to be irradiated with IR. The presented experimental mobility slices are normalized to the corresponding peaks observed in the total quadrupole-selected mobility spectra. Once the data was retrieved for each mobility slice, chromatograms were extracted for each precursor (∑*I*[*nM*]_precursors_^*n*+^) and related fragments (∑*I*[*nM*]_fragments_^*n*+^). The recorded intensity was normalized to 1 s irradiation, meaning that the signal intensity of each extracted *m*/*z* was multiplied by the quotient of 1 s divided by the irradiation time. Each presented IR spectrum is an average over multiple recorded measurements, with the number of obtained IR spectra per mobility peak and per IR region depending on the ion intensity of the precursor over the experimental time. The exact details are summarized in Table S1. Each averaged precursor and fragment signal is used to calculate the photofragmentation yield using the following equation:

$$p\mathrm{hotofragmentation}\;y\mathrm{ield}=-\ln\;\left(\frac{I_{\mathrm{precursor}}}{\left(\sum I_{\mathrm{fragments}}+I_{\mathrm{precursor}}\right)}\right)$$

This calculated photofragmentation yield is plotted as a function of the wavenumber. The wavenumber is determined by correlating the mass spectrometer scan number with the corresponding wavenumber at the moment of the recording, using a classic linear fit (*y* = *a* + *bx*). The resulting spectra are then plotted (dots) and smoothed using a fast Fourier transform (FFT) of 5 points (solid line).

### CCS Calibration

The employed CCS calibration protocol was reported elsewhere.^19,47^ Agilent ESI tuning mix and polyalanine (1 mg mL^–1^ in 50:50 AcN/H_2_O with 1% formic acid) were used as calibrants with instrumental conditions identical to the ones used to measure both peptides. All measurements were performed by using N_2_ as a drift gas in the mobility cell. The arrival time values extracted from each *m*/*z* channel were smoothed in MassLynx, and the values used in the calibration procedure were read from the peak apex. An overview of the calibration data can be found in Table S3 together with the procedure obtained using the set of Excel spreadsheets from the group of Pagel et al. (Freie Universität Berlin). All calculated ^TWIMS^CCS_N2_ values from WT-PD1 and WT-PD2 are reported in Tables S4 and S5.

## Results

### Investigation of WT-PD1 Secondary Structure with IM-MS and IRMPD

The studied wild-type segment of the NACore of α-Synuclein, named here WT-PD1 with sequence _68_GAVVTGVTAVA_78_, is presented in Scheme 1A. Using our modified Photo-Synapt,^18^ a clear peak at a mass over a charge ratio of 944.5 *m*/*z* is observed, originating from the singly charged monomeric ion ([M + H]^1+^). WT-PD1 displays a linear type of oligomerization, meaning a linear increase of both the mass and charge of the ions. This results in the [*nM*]^*nz*+^ type of oligomers (with *n* = number of monomeric units, *M* = protonated monomeric mass, and *z* = charge), where each formed oligomer is measured at the same *m*/*z* channel.

The quadrupole-selected arrival time spectrum of the 944.5 *m*/*z*, presented in Figure 1A (black dashed line), shows two well-resolved peaks at 16.9 and 11.8 ms, corresponding to the singly charged monomer and the doubly charged dimer, respectively, identified using their extracted isotopic distribution as shown in Figure 1B,C. Additionally, a larger feature is present in the mobilogram ranging from 6.6 to 11 ms. The identity of the oligomer responsible for this mobility peak cannot be retrieved from the isotopic pattern directly, as the observed resolution was not sufficient to obtain a clear Δ*m*/*z* (see Figure 1D). This broad feature is typically observed a few hours after sample preparation and is a signature of the aggregation process undergone by WT-PD1.^48^ The experimental recorded mobility slices are colored pink in Figure 1A, representing the [1]^1+^, [2]^2+^, and [*nM*]^*n*+^ oligomers. From these species, mass- and mobility-selected IR fragmentation spectra are recorded and are shown in Figure 2. The IR laser was scanned between 2800 and 3500 cm^–1^ to include both the CH stretch, OH stretch, and the amide A (NH stretch) region. The spectra are highlighted in four different regions in Figure 2, namely, the CH region in gray, the NH_3_^+^ region in green, the OH region in red, and the NH region in blue.

**Figure 1:**
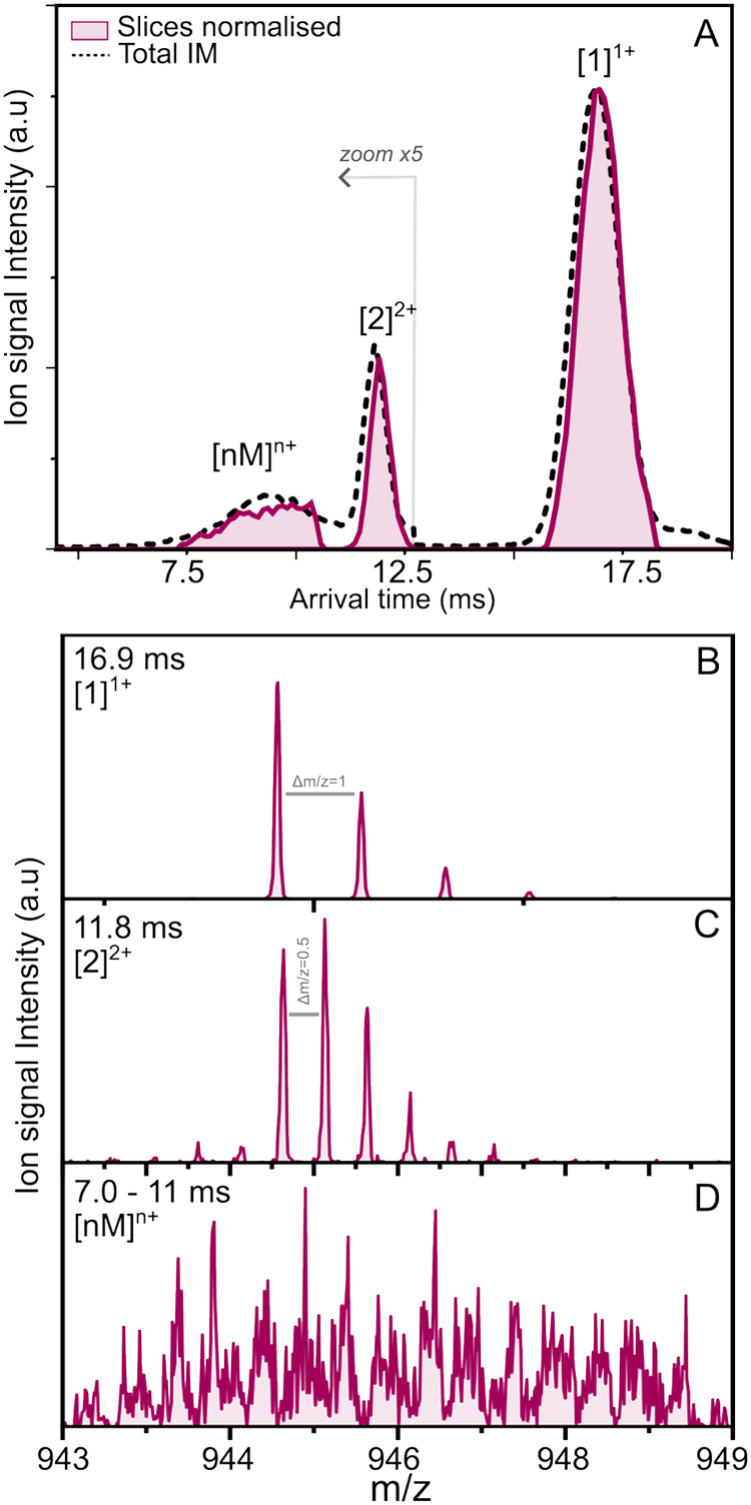
(A) Averaged ion mobility spectrum (dashed line) of quadrupole-selected 944.5 *m*/*z* from WT-PD1. The signal below 12.8 ms is multiplied by 5. Experimental normalized mobility slices of selected ions in pink. (B–D) Extracted mass spectra from each mobility-sliced peak, zoomed on the isotopic distribution of the 944.5 *m*/*z* ion, showing Δ*m*/*z* of 1 for panel (B) corresponding to singly charged ions [1]^1+^, Δ*m*/*z* of 0.5 corresponding to doubly charged ions [2]^2+^ for panel (C), and panel (D) shows a broad peak distribution.

**Figure 2:**
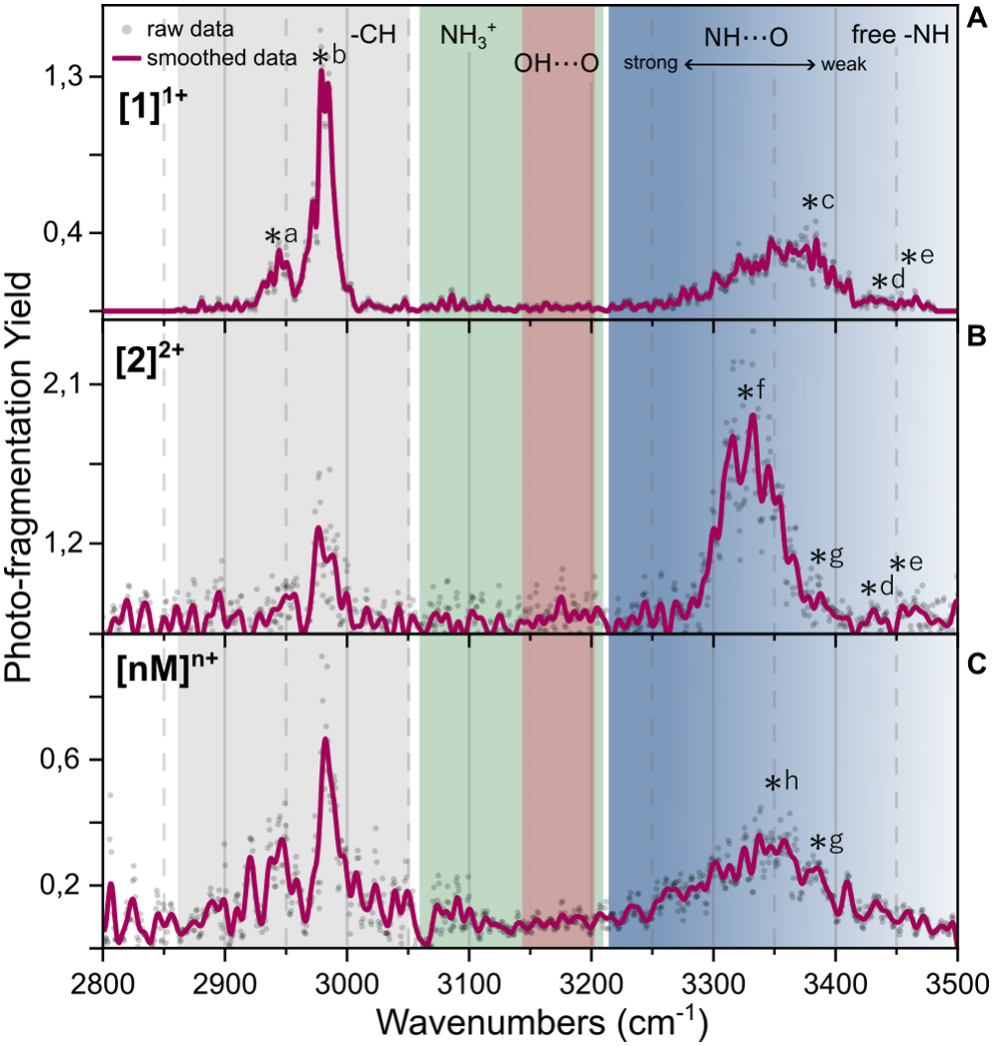
IRMPD spectra of mass- and mobility-selected ions from WT-PD1. (A) Singly charged monomer, (B) doubly charged dimer, and (C) higher-order oligomers of [*nM*]^*n*+^. The gray region highlights the expected CH stretch position, the green NH^3+^, the red H-bonded OH, and in blue, the NH stretches according to the strength of the hydrogen bonds involved (from light to dark corresponding to too weak to strong H-bonding). The black lettered asterisks indicate the peaks discussed in the text.

### Common Vibrational Modes in Protonated PD Peptides

In the CH stretching region (gray), two intense peaks between 3010 and 2915 cm^–1^ are observed corresponding to the antisymmetric and symmetric CH_3_ and CH_2_ modes.^41,49,50^ Below 2915 cm^–1^, a few very small peaks are visible, likely corresponding to additional CH vibrations.^36,51^ The two strong CH peaks are present for the singly charged monomer, the doubly charged dimer, and the higher-order oligomer IR spectrum of WT-PD1. However, as the assembly size increases, the intensity ratio is affected, and we observed a strong increase in the baseline under this specific region. The typical IR signatures of the protonated amide (−NH_3_^+^) are located between 3050 and 3200 cm^–1^ ^36,37^ and tend to be broad and sensitive to the environment.^52^ For the monomer, a weak activity is observed due to its relatively low abundance. The IRMPD spectrum of the larger oligomers (Figure 2C) shows an increase in intensity, coinciding with the increase of the number of protonation sites and thus NH_3_^+^ moieties. Typical free OH stretching vibrations are detected between 3600 and 3800 cm^–1^ and undergo a very large red shift (>300 cm^–1^) when involved in hydrogen bonding (OH···O).^53–55^ Free OH signatures were not observed during our experiments (not shown), indicating that the OH is most likely involved in H-bonding.

### Singly Charged Monomer (Amide A Region) of WT-PD1

Free to very weakly hydrogen-bonded amide A (NH stretch) vibrations of the amide group (−CO–NH–) are typically found between 3420 and 3550 cm^–1^ depending on the amino acids at play.^56^ In the IR spectrum of the singly charged monomer (Figure 2A), the presence of the free or weakly bound NH moieties can be observed by the two weak peaks centered at about 3434 and 3465 cm^–1^ (asterisks d and e). Often, these rather weak signatures are found in β-strands, which are a common secondary structure in peptides. These β-strands are known to stabilize a more extended version of the structure through weak C_5_ interactions.^45^ The broad band between 3265 and 3420 cm^–1^ indicates the presence of additional and stronger intramolecular hydrogen-bonded NH vibrations, resulting from the combination of multiple structural motifs.^42,57,58^ The position of this band toward lower wavenumbers is in agreement with the interaction of the NH moieties with the hydrogen-bond accepting C=O group,^56^ indicating that the peptide is partly folded.

### Doubly Charged Dimer of WT-PD1

The maximum of the NH stretching band (blue in Figure 2B) is red-shifted for the [2]^2+^ ions as compared to that of the [1]^1+^ ions. The shifted peak position, centered at 3330 cm^–1^ (asterisk f), together with the increased intensity, indicates the presence of stronger H-bonded NH groups in the doubly charged dimer. This is consistent with the ordered assembly of two monomeric units. The peaks centered at 3434 and 3465 cm^–1^ (d and e) are preserved from the monomer to the dimer formation, which is in line with the presence of β-strands preserved in larger oligomers.

Comparison of the NH mode signatures to the work of Fricke et al. on dipeptides (see Figure S3), suggests that the stacking of these units follows an antiparallel β-sheet pattern. They observed a characteristic signature of antiparallel β-sheet formation in the range between 3280 cm^–1^ and 3400 cm^–1^, resulting from the presence of three distributed, equidistant peaks as determined using B3LYP/cc-pVDZ calculations.^40^ Under our experimental conditions, this would appear as a broad, potentially shapeless feature. In contrast, a parallel β-sheet conformation would result in three closely spaced peaks in a narrower IR range between 3340 and 3380 cm^–1^, which is not observed.

### Higher-Order Charged Oligomers of WT-PD1

The IRMPD spectrum resulting from the higher-order oligomer [*nM*]^*n*+^ region of the mobility spectrum is shown in Figure 1C. The band in the NH stretching region is weaker in intensity, but covers the wavelength region that combines the signatures observed for [1]^1+^ and [2]^2+^. The maximum of this band is centered between the maxima of the monomer and dimer peaks with a similar width, but the increased baseline makes it appear broader. The elevated baseline suggests unwanted oligomer fragmentation during the trapping stage, while the broad peak indicates vibrational congestion from the large number of NH oscillators, the presence of numerous oligomers, and possible conformers.^41,42^ The NH stretch analysis and IR spectral changes of [1]^1+^, the [2]^2+^, and the oligomer [*nM*]^*n*+^ ions suggest that WT-PD1 oligomers are characterized by an antiparallel β-sheet-type structure.

### Investigation of WT-PD2 Secondary Structure with IM-MS and IRMPD

The pre-NAC wild-type segment _47_GVVHGVATVA_56_ (WT-PD2) shares its amino acid composition with WT-PD1, except for histidine (H or His) at the 50th position, a basic residue possessing an aromatic ring; see Scheme 1B. While neutral at pH 7.4, His can acquire a secondary protonation site when reaching pH < 4.5 under (nano)­ESI conditions. Its imidazole group may influence oligomerization via hydrogen-bonding and proton-sharing interactions.

Singly protonated WT-PD2 ([M + H]^1+^) is detected by mass spectrometry at 909.5 *m*/*z*, and its [*nM*]^*n*+^ oligomers are detected at the same *m*/*z* channel. The arrival time spectrum (dashed line in Figure 3A) of quadrupole-selected (*m*/*z* 909.5) WT-PD2 shows four peaks, of which two appear as a shoulder. The charge states were identified using the respective extracted isotopic distribution observed in the mass spectra; see Figure 3B,G. As indicated by the experimental recorded mobility slices in blue, from right to left in the arrival time spectrum, the most intense peak at 15.9 ms corresponds to the monomer singly charged [1]^1+^. Centered at 12.3 ms is the dimer doubly charged [2]^2+^ with a shoulder belonging to a trimer triply charged [3]^3+^ conformation at 11.6 ms (conformer 1). A second, more compact trimer conformation ([3]^3+^) is observed at 10.2 ms (conformer 2). This [3]^3+^ peak (at 10.2 ms) also presents a shoulder at 9.5 ms, which originates from a quadruply charged tetramer [4]^4+^. Lastly, a broad peak centered at 8.6 ms is observed, originating from an ensemble of higher-order oligomers [*nM*]^*n*+^. Under 7.5 ms, the signal could not be isolated for slicing due to the low abundance of the corresponding ions.

**Figure 3:**
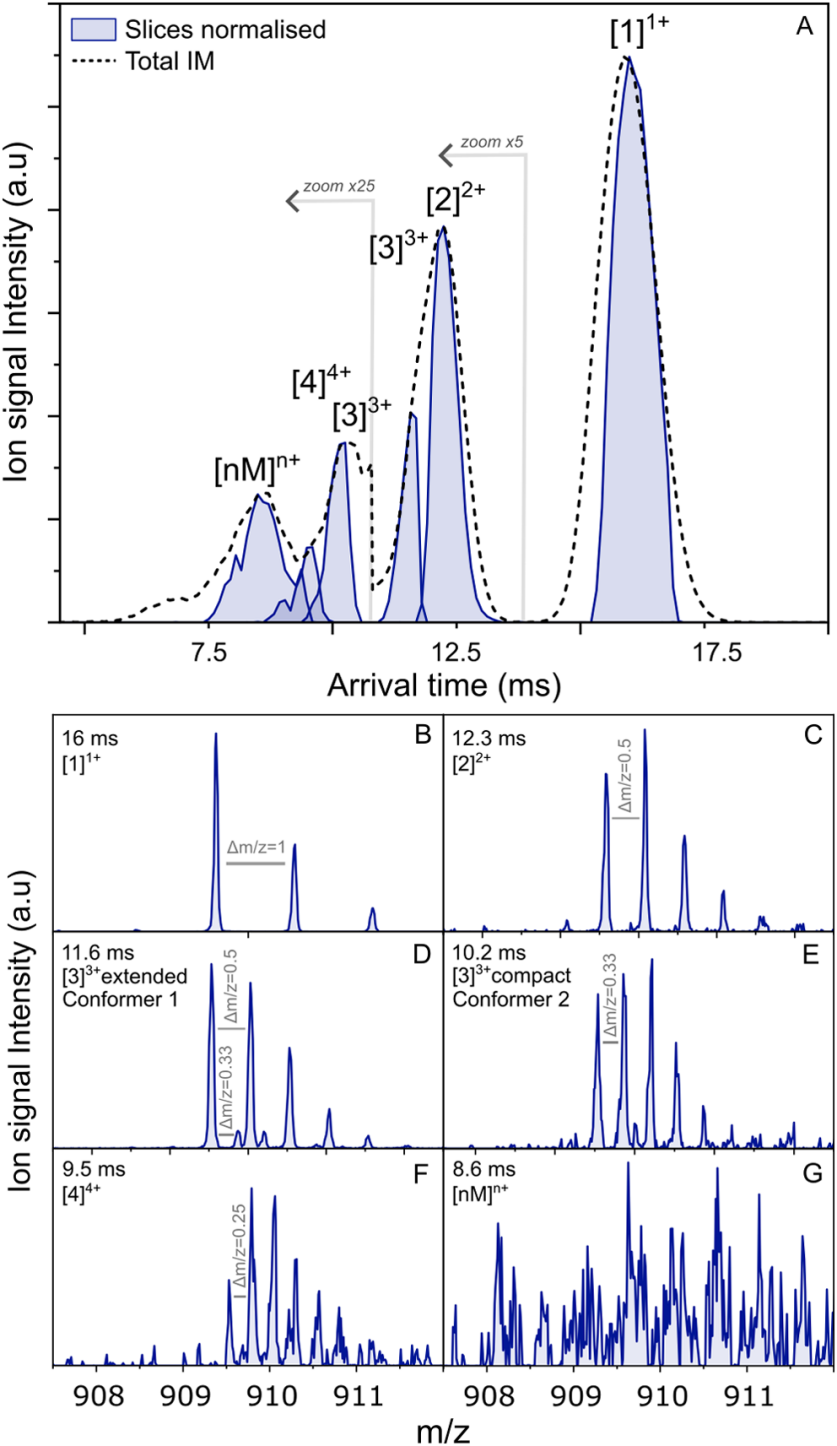
(A) Averaged ion mobility spectrum of quadrupole-selected 909.5 *m*/*z* from WT-PD2 (dashed line); the intensity of the signal was multiplied by 5 under 13.5 ms and by 25 under 10.8 ms. In blue: normalized mobility slices of selected ions. (B–G) Extracted mass spectra from each mobility-sliced peak, zoomed on the isotopic distribution of the 909.5 *m*/*z* ions. (B) Δ*m*/*z* of 1, corresponding to singly charged ions [1]^1+^, (C) Δ*m*/*z* of 0.5, corresponding to doubly charged ions [2]^2+^, (D) Δ*m*/*z* of both 0.5 and 0.33 highlighted with gray lines, corresponding to a combination of [1]^1+^, [2]^2+^, and [3]^3+^ ions and identified as a [3]^3+^ of extended conformation, (E) Δ*m*/*z* of 0.33 highlighted with gray lines, corresponding to triply charged ions [3]^3+^ with a compact conformation, (F) Δ*m*/*z* of 0.25, corresponding to quadruply charged ions [4]^4+^ highlighted with gray lines, and (G) not-resolved distribution.

The presence of a compact and an extended triply charged trimer is not obvious from the total average arrival time spectrum. However, when extracting the mobility spectrum from the *m*/*z* value corresponding uniquely to the [3]^3+^ ions at *m*/*z* 909.9 as displayed in Figure S4 of the SI, we can clearly distinguish two distinct mobility peaks at 11.6 and 10.2 ms. The mass spectrum of conformer 1, Figure 3D (extended conformation), shows an irregular isotopic distribution with contributions of both a strong doubly charged pattern (Δ*m*/*z* = 0.5) and a weak triply charged trimer (Δ*m*/*z* = 0.33). This is no surprise due to the overlapping of the [2]^2+^ and [3]^3+^ mobility peaks, with a contribution of the dimer that is about 10 times more intense. The compact isotopic distribution of the trimer (conformer 2) also does not follow the theoretical isotopic distribution (see Figure S5 of the SI). This results from an additional participation of [1]^1+^ and [2]^2+^ isotopic patterns, which are typically the ions resulting from the fragmentation of [3]^3+^ ([3]^3+^ → [1]^1+^ + [2]^2+^). This indicates that ions undergo spontaneous fragmentation after the ion mobility separation and prior to their arrival at the mass detector, modifying the resulting isotopic pattern in a combination of three different charge states^20,21,59^ and therefore preventing unambiguous identification of the oligomeric ions (labeled as [*nM*]^*n*+^). This is evident in the extracted mass spectrum of the higher oligomers with an arrival time centered at 8.6 ms (Figure 3G), where no distinct Δ*m*/*z* pattern is observed, and additional *m*/*z* signals are detected in the total mass spectrum (Figure S6 of the SI). The isotopic distribution of [4]^4+^, presented in Figure 3F, however, follows the theoretical expectations, indicating a possibly more stable oligomer.

The IRMPD spectra of the [1]^1+^ to [*nM*]^*n*+^ oligomeric ions from WT-PD2 are presented in Figure 4A,E, with two conformers of [3]^3+^ presented in Figure 4C,D. The [4]^4+^ IR spectrum is presented in Figure S7 of the SI. The origin of the observed NH peaks is discussed in the following section. Similarly to WT-PD1, the CH signatures of WT-PD2 for all oligomers are observed at the same IR frequencies as WT-PD1. A more pronounced activity of the main CH_2_ and CH_3_ mode peaks (between 2800 and 3100 cm^–1^) is present for the larger assemblies.

**Figure 4:**
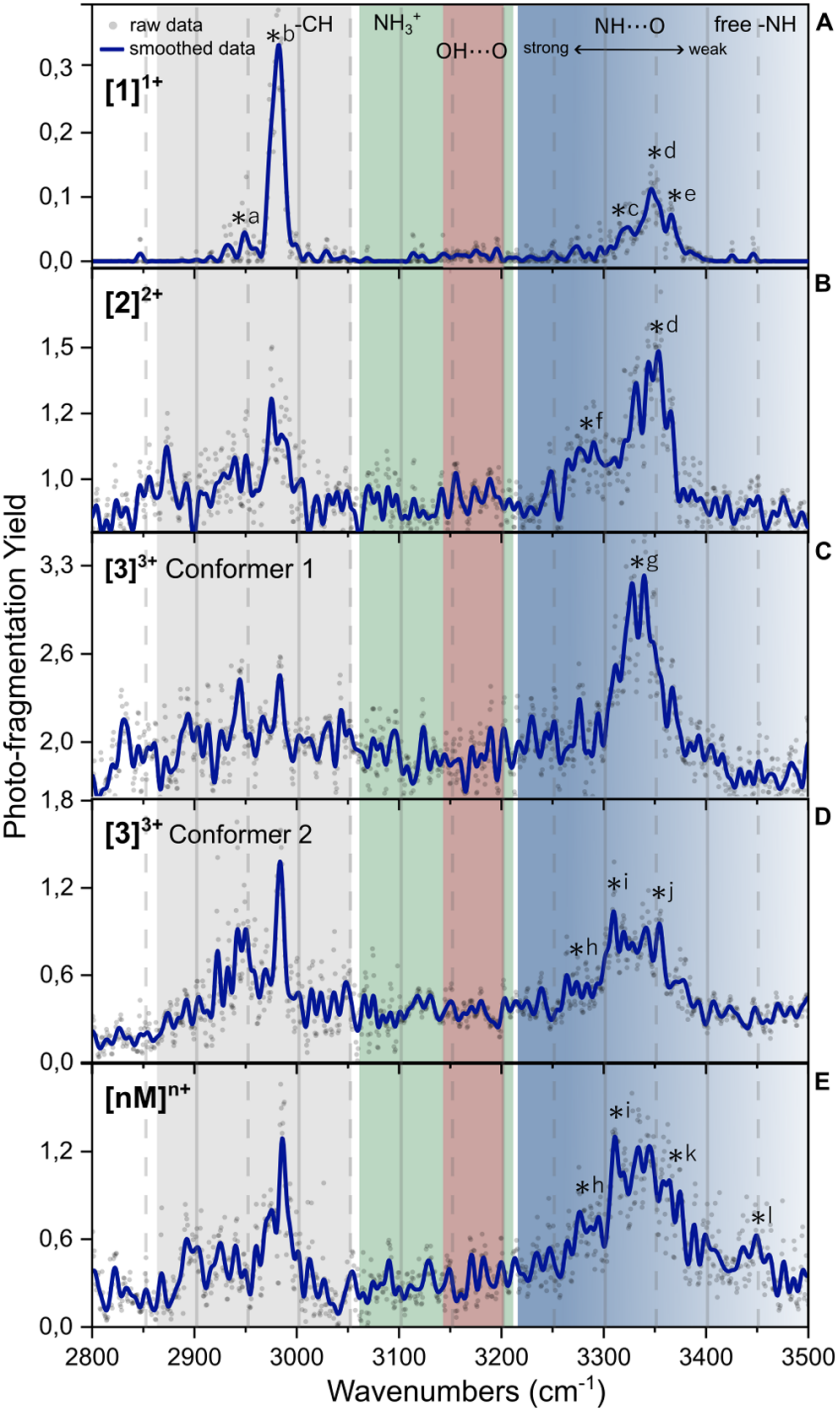
IRMPD spectra of mass- and mobility-selected WT-PD2 ions depicted in Figure 3. (A) Singly charged monomer, (B) doubly charged dimer, (C) triply charged trimer (conformer 1 with extended conformation), (D) triply charged trimer (conformer 2 with compact conformation), and (E) higher-order oligomers of [*nM*]^*n*+^ *m*/*z*. The gray region highlights the CH stretch position, the green NH^3+^, the red OH involved in H-bonding, and in blue the NH stretches according to the strength of the hydrogen bonds involved (from light to dark corresponding from weak to strong H-bonding). The black lettered asterisks indicate the peak positions discussed in the text.

### Singly Charged Monomer (Amide A Region) of WT-PD2

The NH stretching region, indicated in blue in Figure 4, exists as a convoluted peak between 3300 and 3400 cm^–1^ with a maximum centered at 3350 cm^–1^ (asterisk d). The position of this large peak is attributed to H-bonded NH vibrations. Compared to WT-PD1, the H-bond NH peak of WT-PD2 shows a narrower profile, i.e., less extended to the red and blue sides of the main peak, with the center of the peak located at slightly lower wavenumbers. This indicates that the overall structure is more repetitively organized and therefore has a different type of secondary structure than that of WT-PD1. The influence exerted by the aromatic ring from the His residue could be a factor in this difference, as the His side chain can also engage in H-bonds. This more uniform organization (shown as a narrow IR peak), along with the presence of glycine residues in the peptide, is consistent with the possible presence of C_7_-type H-bond interactions, such as γ-turns or 2_7_-ribbons, which are reported between 3250 and 3380 cm^–1^.^40,52^

### Doubly Charged Dimer of WT-PD2

Similar NH signatures between 3300 and 3380 cm^–1^ are observed for the [2]^2+^ ions of WT-PD2 compared to the singly charged monomer, although with increased intensity. Additionally, to the left of this dominant band, a second intense peak extending from 3315 to 3235 cm^–1^ (asterisk f) appeared, which is red-shifted by about 80 cm^–1^. This red shift is consistent with intermolecular NH···O=C hydrogen bonds being formed upon the assembly of the dimer and with the presence of a motif composed of tighter H-bonds, possibly involving the His residue via either H-bond or proton-bound interactions. Since IR activity is still present above 3380 cm^–1^, weakly H-bonded NH groups are also present in the [2]^2+^ structure. The observed IR signatures resemble the pattern of a parallel/γ-turn stacking conformation, previously calculated by Fricke et al.^40^ in the study of the formation of structures of β-sheets in dipeptides. This signature of the parallel/γ-turn conformation is represented by the presence of two bands, with one peak at 3275 cm^–1^ and a combination of peaks between 3350 and 3380 cm^–1^ (determined from B3LYP/cc-pVDZ calculations); see Figure S3.^40^ The distance of approximately 80 cm^–1^ between the peaks allows us to distinguish the two different signatures in our experiments.

### Triply Charged Trimer of WT-PD2: Two Different Conformations

Both conformations of [3]^3+^ were investigated using IRMPD spectroscopy as they could be separated by ion mobility slicing with the Photo-Synapt. Conformer 1 has a later arrival time of 11.6 ms and thus a larger ^TWIMS^ *CCS*_*N*_2__ value of 716 Å (Figure 3C), which indicates an extended conformation. In the NH stretching region of the IRMPD spectra of conformer 1 of the trimer (blue in Figure 4C), a single intense band is observed at 3333 cm^–1^ (asterisk g), similar to that observed for [1]^1+^ and [2]^2+^, but with increased intensity and a red shift of approximately 15 cm^–1^. This is a diagnostic signature of an increased ordered β-sheet structure.^40^ The second conformer of the trimer triply charged is a more compact structure with an arrival time at 10.2 ms and a ^TWIMS^ *CCS*_*N*_2__ value of 662 Å; see Figure 3. The IRMPD spectrum of the compact trimer (Figure 4D) shows a broad, intense peak from 3300 to 3380 cm^–1^ (asterisks i and j) with no apparent maximum, accompanied by a weaker band ranging from 3250 to 3300 cm^–1^. These broad peak shapes indicate the presence of multiple H-bonded NH contributions of different strengths, and the apparent red-shifted indicates that part of these H-bonds is stronger than in conformer 1. This is in agreement with a more compact structure, as was indicated by the smaller ^TWIMS^ *CCS*_*N*_2__ value. The peak shape of the NH modes of conformer 2 resembles the band of the doubly charged dimer, indicating that the [2]^2+^ structure is preserved when the oligomers grow.

### Higher-Order Oligomers of WT-PD2

In the NH stretch region of the IRMPD spectrum of the higher-order oligomers (see Figure 4E), all previously observed features for the smaller assemblies, especially those of the compact triply charged trimer, are preserved. The broad band between 3320 and 3380 cm^–1^ observed for the dimer is further broadened to the low-frequency side (down to 3300 cm^–1^) for the trimer and higher-order [*nM*]^*n*+^ oligomers. The center of the shoulder peak between 3250 and 3300 cm^–1^ (asterisk h) shows a small red shift as well. In addition, we observe an overall broadening of the bands and an increase in the spectrum baseline. This primarily originates from a significant increase in the number of NH oscillators and the close-lying conformation of the oligomeric ions. Both observations indicate a continuous growth of oligomers with the preservation of the previously hypothesized structure, a β-sheet-type structure with a γ-turn dimension. The IR spectrum also shows a peak at the blue side of the spectrum centered at 3450 cm^–1^ (asterisk l), indicating the presence of a weakly bonded NH, which was only visible as very small signatures in the singly charged monomer spectrum (Figure 4A). The combination of weakly and strong hydrogen-bonded NH peaks indicates that only a part of the peptide backbone is involved in β-sheet hydrogen bonding.

### G51D-PD2 Mutant Observed with IM-MS and IRMPD

The G51D mutation from the pre-NAC segment with sequence _47_GVVHDVATVA_56_ (G51D-PD2) results from a substitution of the glycine residue at position 51 by aspartic acid (Asp or D); see Scheme 1C. Aspartic acid has a carboxylate side chain that can be a H-bond donor and acceptor. When negatively charged, Asp is known to destabilize β-sheet formation due to electrostatic repulsion, depending on its position in the sequence. However, in the proximity of a histidine residue, it can favor interactions through salt bridge formation, stabilizing the structure. Singly protonated G51D-PD2 ([M + H]^1+^) is detected by mass spectrometry at 967.5 *m*/*z*, and its [*nM*]^*n*+^ oligomers are detected at the same *m*/*z* channel.

The averaged quadrupole-selected (*m*/*z* 967.5) arrival time spectrum of G51D-PD2 is presented in Figure 5A (dashed line). Three main peaks are observed in the arrival time spectrum at 15.6, 11.5, and 9.4 ms, corresponding to [1]^1+^, [2]^2+^, and [3]^3+^, respectively. The [1]^1+^ and [2]^2+^ peaks were sliced, trapped, and irradiated with IR, from which the spectra are shown in Figure 5B,C, respectively. The [3]^3+^, while intense and well resolved, did not yield sufficient photofragmentation. Similarly, as for the wild types, the CH signatures are still observed at the same IR frequencies and remain constant for the IR action spectra of the G51D-PD2 monomer and dimer; see the gray region of Figure 5B,C.

**Figure 5:**
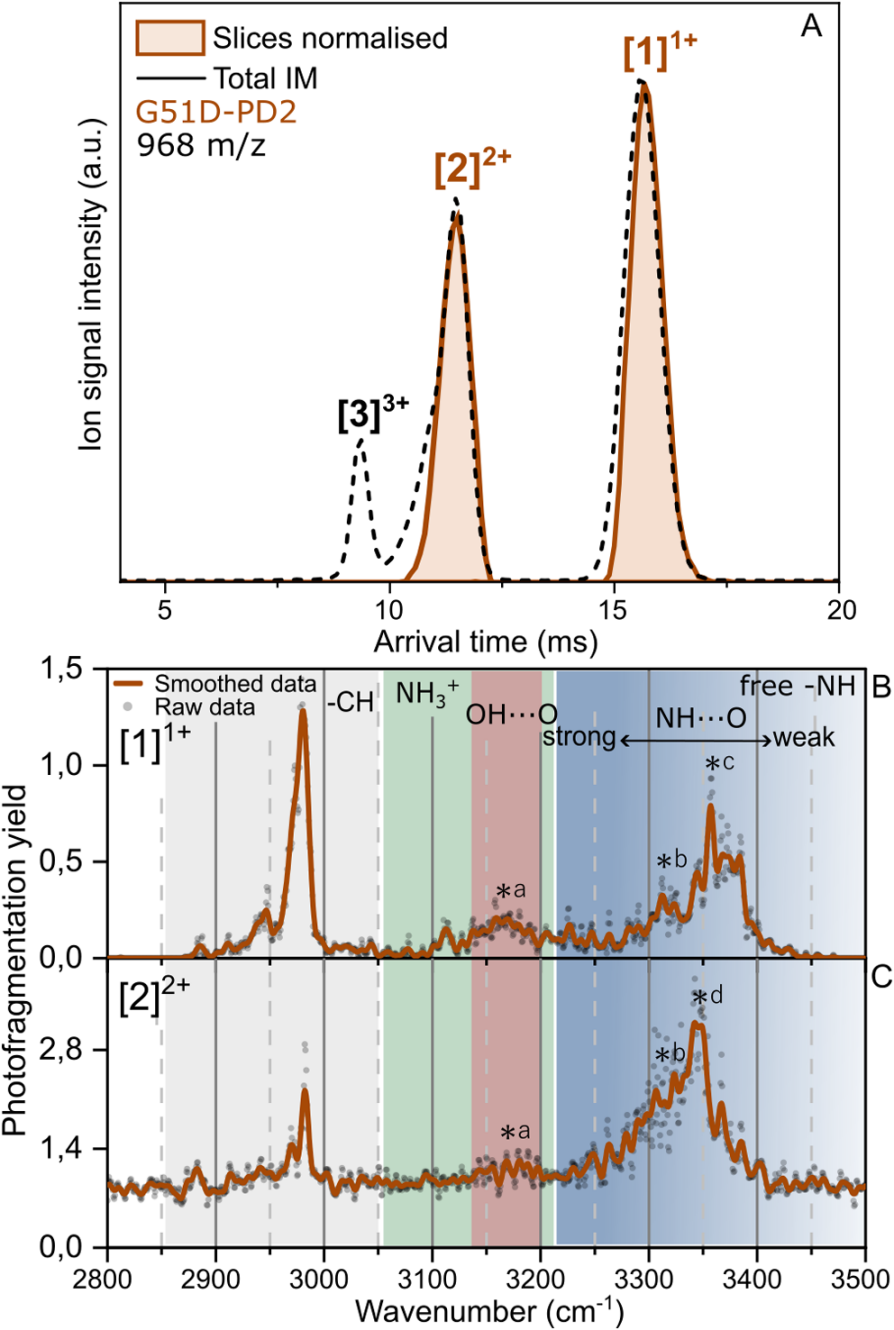
(A) Averaged ion mobility spectrum of quadrupole selected at 967.5 *m*/*z* from G51D-PD2 (dashed line). Experimental, normalized mobility slices of selected ions in orange. IRMPD spectra of mass- and mobility-selected ions from G51-PD2; (B) singly charged monomer; (C) doubly charged dimer. The gray region highlights the expected CH stretch position, the green NH^3+^, the red OH involved in H-bonding, and in blue the NH stretches according to the strength of the hydrogen bonds involved (from light to dark corresponding from weak to strong H-bonding). The black asterisks indicate the peaks discussed in the text.

### Singly Charged Monomer (Amide A Region) of G51D-PD2

The NH stretching region, highlighted in blue in Figure 5, reveals two dominant overlapping features between 3360 and 3440 cm^–1^. The first, observed between 3345 and 3387 cm^–1^ with a maximum at around 3358 cm^–1^ indicated with an asterisk c, corresponds to NH groups involved in moderate hydrogen bonding, while the second, centered at about 3313 cm^–1^ (asterisk b), represents NH groups that are more strongly H-bonded. These observations suggest the presence of tighter structural elements compared to WT-PD1 and similar to those of WT-PD2. The peak at about 3165 cm^–1^ (asterisk a) arises from the additional OH group introduced by the aspartic acid (D) mutation, which is involved in OH···O interactions. The additional OH group enhances the peak intensity compared to that of WT-PD2, which has only two OH groups. This stronger signal between 3150 and 3200 cm^–1^ provides a clear signature of the aspartic acid mutation. As for the other peptide segments, multiple secondary structural motifs are expected to be present. The stronger hydrogen-bond signatures observed for G51D-PD2 align with 2_7_-ribbon structures, typically associated with absorption at lower wavenumbers.^40,52^ This supports the idea of a tighter structure driven by stronger hydrogen bonds.

### Doubly Charged Dimer of G51D-PD2

For the doubly charged dimer ions of G51D-PD2, a convolution of NH vibrations is observed between 3320 and 3410 cm^–1^, which is red-shifted by 30 cm^–1^ and increased in intensity compared to [1]^1+^. This red shift is consistent with NH···O=C hydrogen bonds formed upon assembly of the dimer. The maximum intensity is centered at about 3345 cm^–1^ (asterisk d). This large band shows a slight shoulder at about 3310 cm^–1^ (asterisk b), corresponding to the peak previously observed for the [1]^1+^, indicating that as the order of the assembly increases, a characteristic structural motif from the monomer is preserved in the dimeric [2]^2+^ structure. The band corresponding to OH···O bonded vibrations around 3165 cm^–1^ (asterisk a) is also preserved, although it appears to be attenuated due to the increased baseline.

## Discussion

### IRMPD Highlights Aggregation Patterns through Amide A Vibrations

A comparison between WT-PD2 (_47_GVVHGVATVA_56_), the G51D-PD2 mutant (_47_GVVHDVATVA_56_), and WT-PD1 (_68_GAVVTGVTAVA_78_) reveals distinct secondary structures despite sequence similarities, both at the singly charged monomeric level and in early-stage aggregates. These observations are unique to the application of Photo-Synapt, which allows us to select and investigate unique singly oligomer conformations with IRMPD spectroscopy. While ^TWIMS^ *CCS*_*N*_2__ differences among monomers are minor (<3%, max 8 Å^2^; Figure 6A–C), their IR spectra show unique signatures, particularly in the H-bonded NH stretching region. WT-PD2 and G51D-PD2 exhibit narrower, red-shifted NH bands compared to those of WT-PD1, indicating a more compact structure with more uniform H-bonded NH groups. WT-PD1 shows more activity above 3400 cm^–1^, characteristic of the weakly H-bonded or free NH, supporting a less compact conformation. The clear peak at 3310 cm^–1^ in G51D-PD2 suggests stronger NH bonding, while the peak at 3165 cm^–1^ is associated with H-bonded OH vibrations, which is barely present in the wild-type WT-PD2. This highlights the effect of Asp substitution on the secondary structure of the G51D-PD2 monomer.

**Figure 6:**
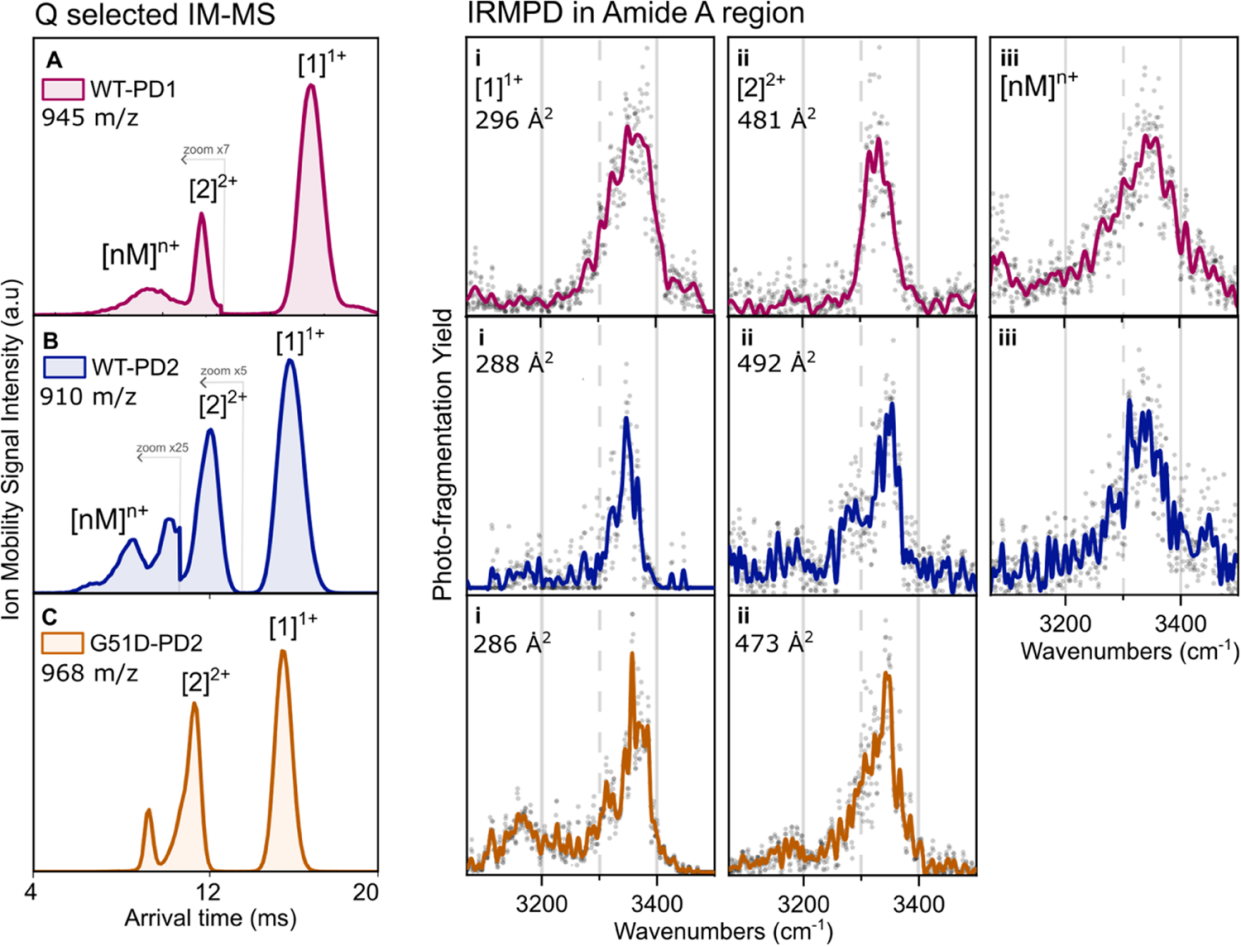
Averaged mobility spectra of (A) WT-PD1, (B) WT-PD2, and (C) G51D-PD2. (i), (ii), and (iii) IRMPD spectra of the singly charged monomer, the doubly charged dimer, and the oligomer ions, respectively, zoomed in on the amide A region, for WT-PD1 (pink), WT-PD2 (blue), and G51D-PD2 (orange) for [1]^1+^ and [2]^2+^.

Large molecules, such as the peptides studied here, often exhibit spectral congestion when studied by IRMPD spectroscopy in the gas phase due to various factors. These include the presence of multiple thermally populated states that are not fully controlled by the collision with the cooling gas in the trap, the presence of multiple conformations, and possible overlapping bands from the diverse functional groups in the peptide sequence.^41,60,61^ This phenomenon is already observed for the monomers in our experiment and only becomes more prominent upon aggregation. Consequently, assigning individual bands is almost impossible, even using a high level of computational methods.^41–43,55^ However, shifts and characteristic combinations of peaks in the amide A region do reveal unique structural signatures.

A comparison between the dimers of WT-PD1 (NAC), WT-PD2 (pre-NAC), and the G51D-PD2 mutant highlights the impact of sequence differences and bulkier residues upon oligomerization. Despite similar ^TWIMS^ *CCS*_*N*_2__ values, their doubly charged dimers exhibit distinct IR signatures, which would remain hidden by IR spectroscopy or IM-MS alone; see Figure 6. WT-PD1 (^TWIMS^ *CCS*_*N*_2__: 481 Å^2^) shows a red-shifted 3330 cm^–1^ peak, suggesting an antiparallel β-sheet. WT-PD2 (492 Å^2^) features two bands at 3350 and 3280 cm^–1^, consistent with a parallel β-sheet with a γ-turn stacking.^40^ Notably, the dimer of WT-PD2 is bulkier than that of WT-PD1, reversing their monomeric trend. The G51D-PD2 dimer (Figure 6C,ii) exhibits a unique spectral profile due to Gly → Asp substitution. Its IR spectrum features a broad band at 3358 cm^–1^ with a 3313 cm^–1^ shoulder, alongside H-bonded OH vibrations, which are absent in the wild type. This mutation induces structural compaction, reducing the ^TWIMS^ *CCS*_*N*_2__ values (−2 Å^2^ for [1]^1+^ and −19 Å^2^ for [2]^2+^). The G51D-PD2 oligomers ([2]^2+^ and [3]^3+^) are more abundant than their WT counterparts, indicating enhanced stability, likely due to His–Asp interactions. Their compact structure resists ion heating, preventing oligomer fragmentation due to ion heating, resulting in strong IR hydrogen-bond signatures and prolonged IRMPD irradiation times (Table S1 of the SI). Minimal structural rearrangement upon dimerization further supports its stability.^62^

Overall, this hyphenated approach allows us to distinguish between different motifs of β-sheets and to identify the potential involvement of additional motifs with unique signatures, such as the presence of β or γ-turns within the sheets.^40^ While this study demonstrates the potential of the amide A region, the assignment could not be achieved without ambiguity.

## Conclusions

In this article, we demonstrate the feasibility and value of using ion mobility mass spectrometry (IM-MS) coupled with IRMPD spectroscopy to analyze the secondary structure of large peptides and their aggregates. The ion mobility dimension enables the separation of oligomers and conformers. Moreover, it provides information about their overall shape (more compact or extended) by determining their ^TWIMS^ *CCS*_*N*_2__ values. The separation of oligomers is crucial to obtain oligomer-specific IR spectra that reveal unique IR signatures to elucidate their secondary structures. Our results, particularly in the amide A region, prove to be diagnostic for characterizing peptide aggregation, even in the absence of high-level quantum chemical calculations. The experimental results demonstrate that by using IR action spectroscopy of mass- and mobility-selected oligomers, we can distinguish between β-sheet and non-β-sheet structures. Furthermore, our approach has the potential to further classify non-β-sheet components, such as β or γ-turns, providing deeper insights into the structure of the monomers and oligomers present in the aggregation process. The comparison between WT-PD1 from the NAC region and WT-PD2 and the G51D-PD2 mutant from the pre-NAC region of α-Synuclein highlights the influence of the peptide sequence and the presence of bulky residues in the oligomerization process, despite the overall sequence similarity. The information obtained in this study characterizes both the initial monomeric folding and the aggregation patterns of each peptide. These mass- and mobility-selected IR features correlate with structural organization, mutation effects, and oligomer stability, providing a unique view of hydrogen-bonding networks and folding dynamics during early aggregation. Extending the IR range to the amide I and II regions (C=O stretch and NH bend) can provide more structural information on the β-sheet structures formed and reduce assignment ambiguities, which is planned for the near future.

## Supplementary Material



## Data Availability

The data underlying this study are openly available in DataCite Commons at https://doi.org/10.48338/VU01-CYMCB7.

## Notes

Additional experimental details, materials and methods (Section S1); additional information for the IM-MS and IRMPD analysis including calculated IRMPD spectra of 3 different β-sheet motifs, arrival time spectrum from Photo-Synapt of quadrupole-selected 910.5 *m*/*z* from WT-PD2 showing the trimer conformation overlapping with the dimer, calculated isotopic distribution MS for WT-PD2 [3]^3+^ and [4]^4+^, total mass spectrum of WT-PD2 mobility slice 8.6 ms, quadruply charged tetramer IR action spectrum from WT-PD2, and replicates of IRMPD spectra in the free NH region for WT-PD1 and WT-PD2 singly charged monomers. (Section S2) (PDF)
