# Supplementary material for: IR Action Spectroscopy of Mass- and Mobility-Selected α‑Synuclein Segments: Structure Identification in Early-Stage Oligomers via the Amide A Region
Source: J Phys Chem A. 2025 Aug 21;129(35):8064–74. doi: 10.1021/acs.jpca.5c02819 (PMC12415830; doi:10.1021/acs.jpca.5c02819)
Supplement: Supplementary file 1 [file jp5c02819_si_001.pdf]

Supplementary Information

IR Action Spectroscopy of Mass and Mobility Selected α-Synuclein Segments: Structure Identification in Early-stage Oligomers via the Amide A Region

Agathe Depraz Depland[a,b], Olivier Verhoev[a], Steven Daly[c] and Anouk M. Rijs[a,b]*

[a] Division of Bioanalytical Chemistry, Department of Chemistry and Pharmaceutical Sciences, Amsterdam Institute of Molecular and Life Sciences, Vrije Universiteit Amsterdam, De Boelelaan 1105, 1081 HV Amsterdam, the Netherlands
[b] Centre for Analytical Sciences Amsterdam (CASA), Amsterdam, the Netherlands
E-mail: a.m.rijs@vu.nl
[c] MS-Vision, Televisieweg 40, 1322 AM Almere, Netherlands

Table of Contents

S1 Experimental Section S2

Table S1 - Experimental conditions of IRMPD experiments S2

Table S2 - Instrumental method parameters for the Photo-Synapt S4

Figure S1 - Ion Mayden parameter file for transmission mode S5

Figure S2 - Ion Mayden file for IRMPD experiments S5

Table S3 - Calibration spreadsheet for the CCS values S6

Table S4 – Calibrated CCS values of WT-PD1 S7

Table S5 - Calibrated CCS values of WT-PD2 S7

S2 Additional information for the IM-MS and IRMPD analysis S8

Figure S3 – Calculated IRMPD spectra of 3 different β-sheet motifs S8

Figure S4 – Arrival time spectrum from photo-Synapt of quadrupole selected 910.5 *m/z* from WT-PD2 showing the trimer conformation overlapping with the dimer S8

Figure S5 – Calculated isotopic distribution MS for WT-PD2 $[3]^{3+}$ and $[4]^{4+}$ S9

Figure S6 – Total mass spectrum of WT-PD2 mobility slice 8.6 ms S10

Figure S7 - Quadruply charged tetramer IR action spectrum from WT-PD2 S10

Figure S8 – Replicated IR action spectra from WT-PD1 singly charged monomers S11

Figure S9 – Replicated IR action spectra from WT-PD2 singly charged monomers S12

S1 Experimental Section

Table S1 - Experimental conditions of IRMPD experiments

	Mobility slice (ms)	Precursor mass (m/z)	Fragments mass (m/z)	IR range (cm^{-1})	Irradiation (ms)	Trap pressure (mbar)	#
**WT-PD1**	15.2	944.3 – 947.3	855 (b_{10}); 837 (b_{10} – H_2O); 756 (b_9) ; 738 (-H_2O)	2800-3500	1200	2.5E-6	2
				3100-3500	1200		1
				3100-3500	1000		1
				2800-3500	800		2
	10.8	944.8 ; 945.8	944.3 ; 945.3 ; 946.3	3100-3500	220	6.7E-6	7
				2800-3500	230		1
				2800-3100	250		5
	7.0	941 - 950	951 – 2500	2800-3500<br>3100-3500	220	5.9E-6	1<br>1
				2800-3500<br>3100-3500	230	5.9E-6	1<br>6
				3100-3500	240	8.7E-6	4
**WT-PD2**	14.6	909.3	820 (b_9) ; 802.5 (-H_2O)	2800-3500	1000	2.5E-6	2
				2800-3500<br>3100-3500	1200	2.5E-6	2<br>2
	11.0	909.8 ; 910.8	909.3 ; 910.3 ; 911.3	2800-3100	175<br>180<br>200<br>300	8.4E-6<br>8.4E-6<br>8.4E-6<br>8.4E-6	2<br>1<br>1<br>1
				3100-3500	100<br>105<br>130	8.4E-6<br>8.4E-6<br>8.4E-6	2<br>6<br>3
	10.7	909.6 ; 909.9 ; 910.6 ; 910.9	909.3 ; 909.8 ; 910.3 ; 910.8	2800-3100	100<br>150<br>165	6.5E-6<br>1E-5<br>1E-5	1<br>1<br>3
				3100-3500	100<br>110<br>120<br>150	8.25E-6<br>1E-5<br>1E-5<br>1E-5	2<br>5<br>2<br>1
	9.6	909.6 ; 909.9 ; 910.6 ; 910.9	909.3 ; 909.8 ; 910.3 ; 910.8	3100-3500	100<br>110<br>130<br>150	8.9E-6<br><br>5E-6<br>5E-6	6<br>2<br>1<br>1
				2800-3100	150	8.9E-6	3
	8.2	909.5 ; 910 ; 910.5 …	909.78; 910.78 ….	3100-3500<br>2800-3500	100<br>110	8.7E-6	1<br>1
	7.2	907.3 – 912.3	754-800 (y_8,a_9)<br>815-825 (b_9)	2800-3500<br>2800-3500<br>3100-3500	240<br>320<br>320	6.2E-6<br>6.2E-6<br>8E-6	1<br>1<br>2

				2800-3100		8E-6	1
**G51D-PD2**	9.0	967.5 – 970.5	949 (MHH2O)<br>878 (b9)<br>860 (b9-H2O)	2700–3110<br>2700–3800<br>3100–3500	1000<br>1800<br>1800	2,8E-6	1<br>5<br>5
	6.9	968 ; 969 ; 970	967,3 ;<br>968,3 ;<br>969,3	2700–3800<br>2800–3600<br>3600–3800	190<br>190<br>190	8,5E-6	1<br>4<br>4
	6.2	968 ; 969 ;<br>970	967,3 ;<br>968,3 ;<br>969,3	2700–3800<br>2800–3110<br>3100–3500	190<br>210<br>190	8,8E-6	3<br>4<br>4
	5.3	967,8 ;<br>968,2<br>968,8 ;<br>969,2	967,3 ;<br>967,8 ;<br>968,3 ;<br>968,8	2700–3800<br>2700–3110<br>3100–3500	145<br>185<br>145	8,9E-6	1<br>7<br>7

Table S1 - Details of IRMPD experiments for both WT-PD1 and WT-PD2 summarising the fragments masses, the irradiation range of frequency, the irradiation time for each recording, the trap pressure associated, and the number of time the experiment was repeated for each mass and mobility selected slice.

Table S2 - Instrumental method parameters for the Photo-Synapt

Polarity	ES+
Analyser	Resolution Mode
Capillary (kV)	Variable
Source Temperature (°C)	80
Sampling Cone	15
Extraction Cone	3.5
Source Gas Flow (mL/min)	0.00
Desolvation Temperature (°C)	150
Cone Gas Flow (L/Hr)	4.0
Purge Gas Flow (mL/h)	400.0
Desolvation Gas Flow (L/Hr)	400.0
LM Resolution	4.9
HM Resolution	15.0
Aperture 1	0.0
Pre-filter	2.0
Ion Energy	1.0
Trap Collision Energy	4.0
Transfer Collision Energy	0.0
Trap Gas Flow (mL/min)	1.00
Helium Cell Gas Flow	200.00
IMS Gas Flow (mL/min)	45.00
Collision Energy	4.0
Trap DC Entrance	3.0
Trap DC Bias	32.0
Trap DC	-2.0
Trap DC Exit	2.0
IMS DC Entrance	15.0
Helium Cell DC	20.0
Helium Exit	-3.0
IMS Bias	3.0
IMS DC Exit	0.0
Transfer DC Entrance	0.0
Transfer DC Exit	2.0
Source Wave Velocity (m/s)	200
Source Wave Height (V)	0.2
Trap Wave Velocity (m/s)	313
Trap Wave Height (V)	6.0
IMS Wave Velocity (m/s)	260
IMS Wave Height (V)	11.0
Transfer Wave Velocity (m/s)	191
Transfer Wave Height (V)	4.0
Mobility Trapping Release Time (μs)	200
Mobility Trap Height (V)	15.0
Mobility Extract Height (V)	0.0

Acquisition mass range	
Start mass	150.000
End mass	2500.000

Table S2 - Instrumental parameters from the Photo-Synapt experiments

Figure S1 - Ion Mayden parameter file for transmission mode

Ion Mayden is the software in charge of the voltages and other parameters relative to the customized part of the instrument. More details to be found elsewhere.[3]

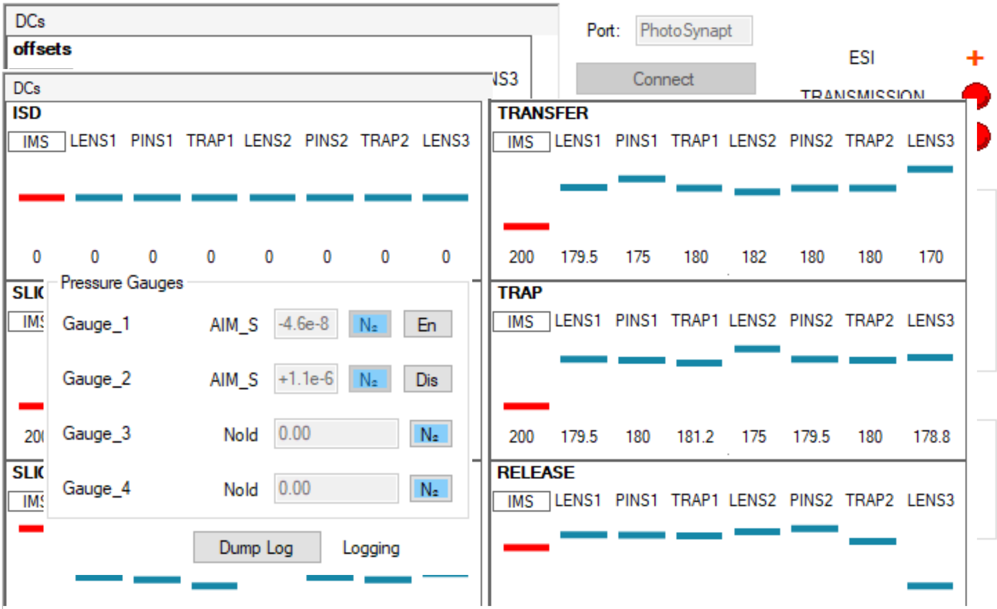

Figure S1 - Ion Mayden parameters for the positive transmission mode with ion mobility on. Transmission of positive ions.

Figure S2 – Ion Mayden parameters for the slicing of ions from mobility and subsequent trapping for IR irradiation of the ions.

Figure S2 - Ion Mayden file for IRMPD experiments

With HEX1 -178,75 V and HEX2 -179,5 V and the pressure varied depending on the nature of the trapped ions. Those values are summarised in the detailed experimental parameters for the IRMPD experiment Table S2.

Table S3 - Calibration spreadsheet for the CCS values

(https://www.bcp.fu-berlin.de/en/chemie/chemie/forschung/OrgChem/pagel/research/carbohydrates/index.html)

**EDC delay coefficient**			90,00	ms			
**Drift gas mass**			28	Da			
**substance**	**m/z**	**z**	**MW in Da**	**lit. CCS in Å²**	**dt in ms**	**logarithmic fit**	
						**ln(CCS')**	**ln(dt')**
**PolyAla**	516	1	515	211,00	8,6	6,99	1,88
	1014	1	1013	306,00	17,86	7,38	2,71
	658	1	657	243,00	11,25	7,14	2,19
	720	2	1438	404,00	8,49	6,96	1,80
	801	1	800	271,00	13,78	7,25	2,42
	826	2	1651	437,00	9,81	7,04	1,98
	943	1	942	294,00	16,54	7,34	2,62
**TuneMix**	622	1	622	202,9	8,27	6,96	1,80
	922	1	922	243,6	11,91	7,15	2,22
	1222	1	1222	282,2	15,88	7,30	2,54
	1522	1	1522	316,9	19,84	7,42	2,79

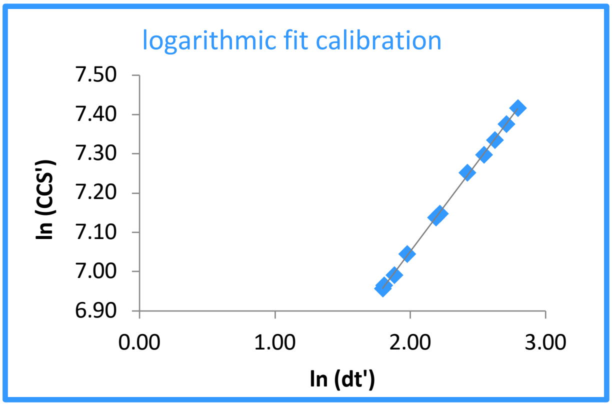

Logarithmic Fit

ln(CCS')=x ln(dt') + ln A

x	0,4585
lnA	6,1343
A	461,4346
R^2	0,99953

Table S3 – Table with the data necessary for the calibration of CCS values from the Photo-Synapt.The logarithmic fit is selected as the best fit against the linear one, and the calculated parameters for the fit from the calibration values are presented in blue.

As described in the method section, a selection of Poly-Alanine ions and Agilent TuneMix ions were selected to calibrate the CCS values for WT-PD1 and WT-PD2 These are presented in this Table S3, as well as the logarithmic fit selected and its corresponding parameters. Due to the customisation of the instrument, the ions path length is modified between the mobility cell and the ToF mass detector. Therefore, the drift time of the ions need to be corrected for. This requires the determination of an empirical constant named

the enhanced duty cycle (EDC). This was estimated by measuring the drift time of tuning mix and denatured ubiquitin, cytochrome C and myoglobin, and reported by Stroganova et. al[1]. In our case, the optimal EDC for the experimental settings reported here was found to be 90 with a minimum error was approximately 1 %. The full length of the calibration procedure and theory behind it was also described in detail by Stroganova *et. al.*[1]

Table S4 – Calibrated CCS values of WT-PD1

The CCS values calculated from the mobilities of WT-PD1 obtained using the TIMS-Qq-ToF From Bruker, and the ones from the Photo-Synapt are very similar. Mobilities were measured using N_2 as a drift gas.

m/z	z	N	MW in Da	dt in ms	log fit CCS* in Å²	TIMS CCS in Å²
944,3	1	1	943,30	16,87	**297,76**	293,9
944,3	2	2	1886,60	11,80	**481,99**	477,4

Table S4 – CCS values of WT-PD1 calculated from the arrival times measured with the Photo-Synapt using logarithmic fit. TIMS CCS values measured from are presented for comparison

Table S5 - Calibrated CCS values of WT-PD2

m/z	z	N	MW in Da	dt in ms	log fit CCS* in Å²	TIMS CCS in Å²
909,54	1	1	908,50	16,00	**289,87**	290,7
909,54	2	2	1817,00	12,20	**493,03**	499,1
909,54	3	3	2725,50	11,60	**715,90**	733,6
909,54	3	3	2725,50	10,20	**661,78**	666,3
909,54	4	4	3634,00	9,50	**842,46**	n/a

Table S5- CCS values of WT-PD2 calculated from the arrival times measured with the Photo-Synapt using logarithmic fit. TIMS CCS values measured from are presented for comparison

The CCS values calculated from the mobilities of WT-PD2 obtained using the TIMS-Qq-ToF From Bruker, and the ones from the Photo-Synapt are very similar. Mobilities were measured using N_2 as a drift gas.

S2 Additional information for the IM-MS and IRMPD analysis

Figure S3 – Calculated IRMPD spectra of 3 different β-sheet motifs

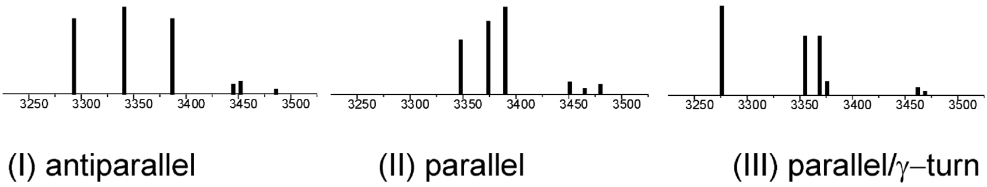

Figure S3 - The calculated stick spectra in the Amide A region of (Ac-Val-Tyr(Me)-NHMe)$_2$ represent the most stable types of antiparallel (I), parallel (II), and parallel/γ-turn (III) conformers at the (a) B3LYP/cc-pVDZ level of theory. Reprinted and adapted with permission from Fricke et al. from ref[5]. Copyright 2024 from American Chemical Society.

Figure S4 – Arrival time spectrum from photo-Synapt of quadrupole selected 910.5 *m/z* from WT-PD2 showing the trimer conformation overlapping with the dimer

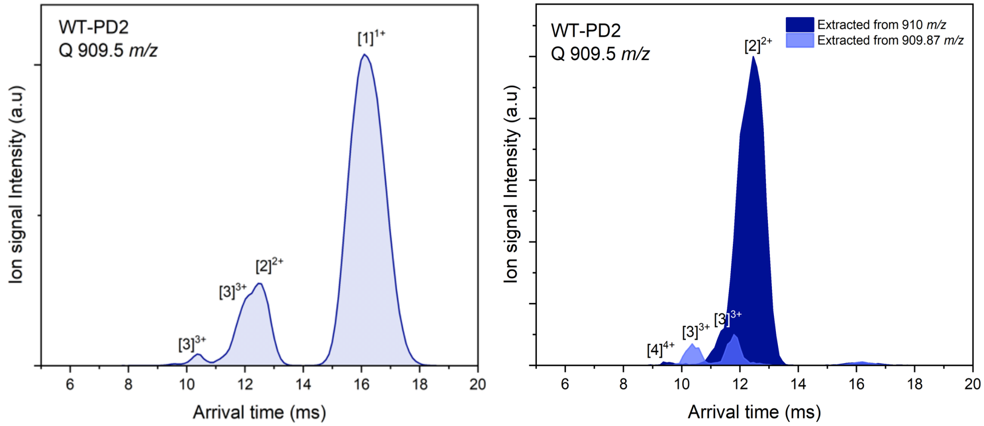

Figure S4 – On the right the total ion mobility spectrum of the Q selected m/z 909,5 from WT-PD2 peptide, at an arbitrary point in time. On the left, superposition of two mobility spectra extracted from : In dark blue, the m/z 910 corresponding to the isotopic peak of the doubly charged dimer $[2]^{2+}$ and potentially $[4]^{4+}$. In lighter blue, the m/z 909,87 corresponding to the trimer triply charged isotopic peak $[3]^{3+}$.

This figure shows the presence of an obvious shoulder on the left side of the $[2]^{2+}$ peak from the total arrival time spectrum of the quadrupole selected 909.5 *m/z*. This emphasize the presence of two conformers of $[3]^{3+}$ previously discussed. When extracted from the isotopic peak of the MS corresponding to the $[3]^{3+}$, to separated peaks of equivalent intensity are observed corresponding to two distinct conformers.

Figure S5 – Calculated isotopic distribution MS for WT-PD2 [3]$^{3+}$ and [4]$^{4+}$

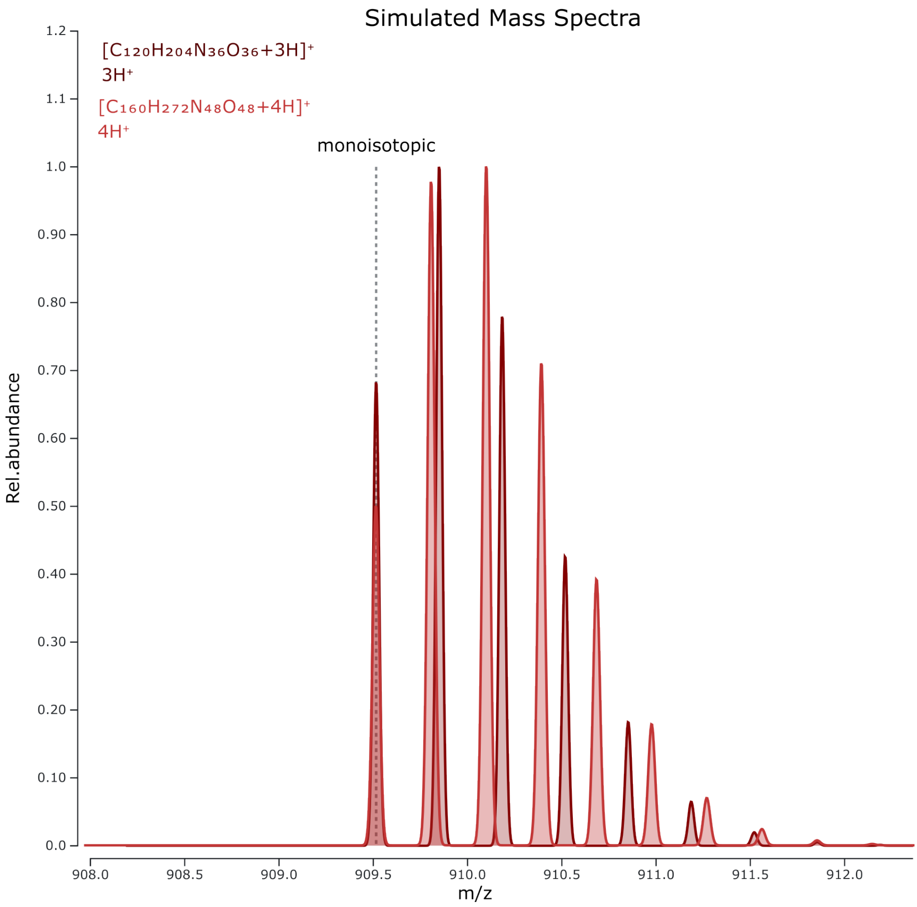

Figure S5 – Simulated mass spectra of the theoretical isotopic distribution of the triply charge trimer (dark red) and the quadruply charged tetramer (light red) of WT-PD2. © 2014 - 2025 | Prot pi. Last consulted on January 13th 2025.

Figure S6 – Total mass spectrum of WT-PD2 mobility slice 8.6 ms

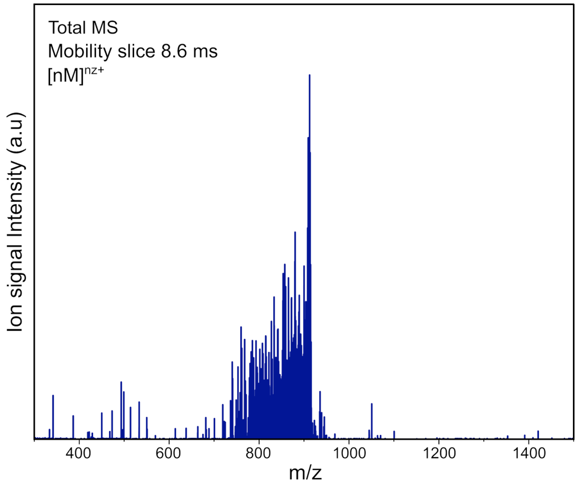

Figure S6 – Total mass spectra of Q selected 909.5 m/z from WT-PD2 sliced in mobility at arrival time 8.6 ms corresponding to oligomers mobility.

Figure S7 - Quadruply charged tetramer IR action spectrum from WT-PD2

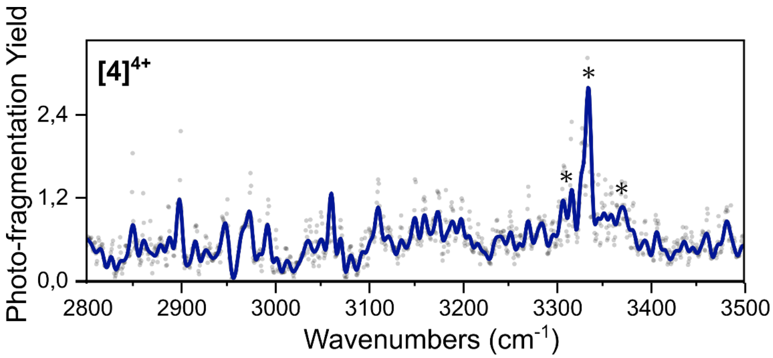

Figure S7 - IRMPD spectrum of the quadruply charged tetramer ions from WT-PD2.

The quadruply charged tetramer was very short lived and did not allow for a sufficient amount of IR spectra to be recorded before disappearing. Here only two spectra were averaged, one covering the full 2800 to 3500 cm^{-1} range and the second one only the 3100 to 3500 cm^{-1} range. In agreement with previous observations, the IR action spectrum of the $[4]^{4+}$, presented shows similar features as the $[2]^{2+}$ and compact $[3]^{3+}$ in the NH stretch region between 3245 and 3280 cm^{-1}, although the broad peak is dominated by a sharp peak at 3335 cm^{-1} that probably originates from signal instability and a lack of data in the average. An increase of activity is observed over the full IR range.

Figure S8 – IR action spectra from WT-PD1 singly charged monomers illustrating reproducibility of NH stretch region

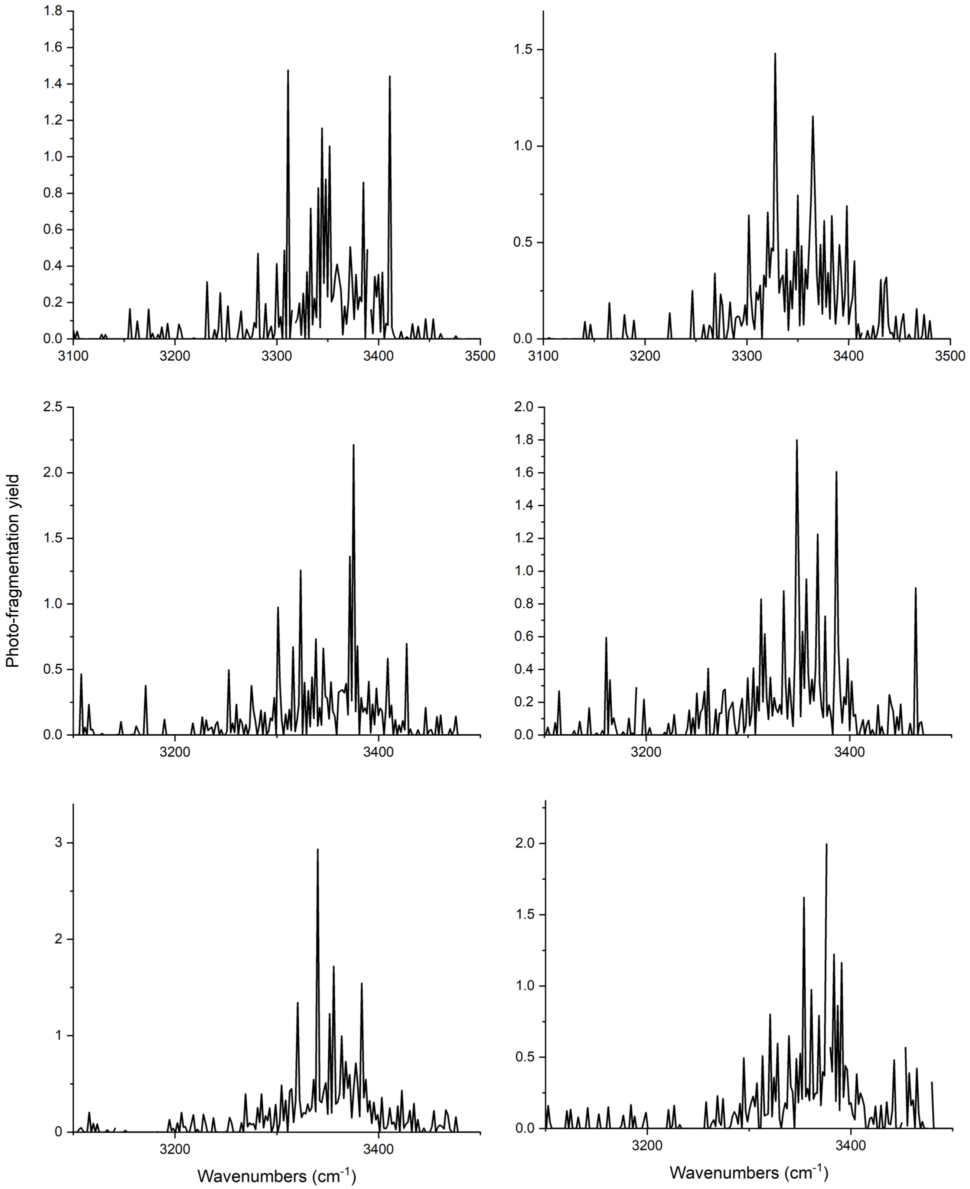

Figure S8 – Six replicates of the IRMPD spectra of the singly charged monomer of WT-PD1 focused on the free NH region from 3100 to 3500 cm^{-1} showing activity for all individual scans (even when intensity is low).

Figure S9 – IR action spectra from WT-PD2 singly charged monomers illustrating reproducibility of NH stretch region

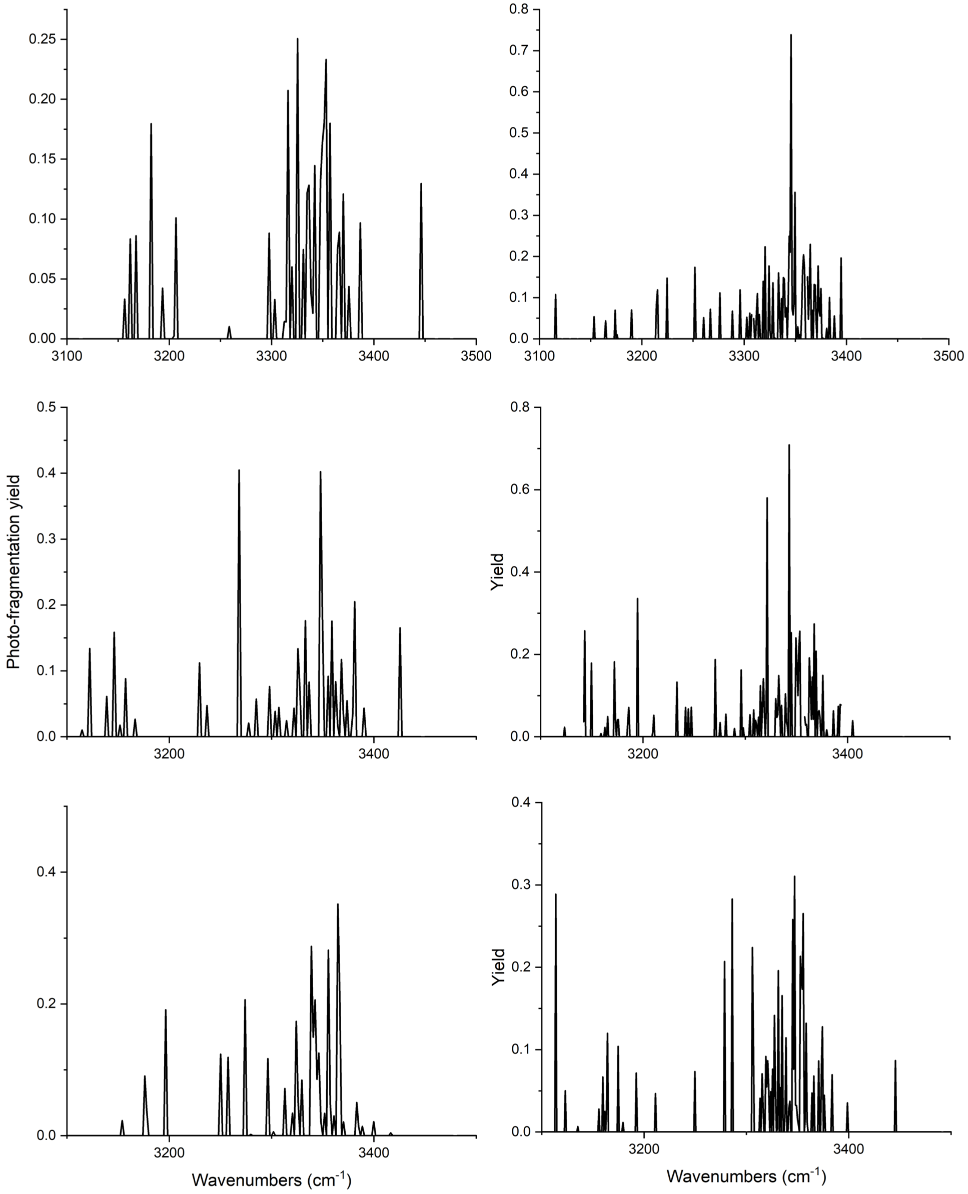

Figure S9 - Six replicates of the IRMPD spectra of the singly charged monomer of WT-PD2 focused on the free NH region from 3100 to 3500 cm^{-1} showing activity for all individual scans (even when intensity is low).
